# Photodegradation and adsorption of hexazinone in aqueous solutions: removal efficiencies, kinetics, and mechanisms

**DOI:** 10.1007/s11356-022-19205-y

**Published:** 2022-02-21

**Authors:** Tahereh Jasemizad, Lokesh P. Padhye

**Affiliations:** Department of Civil and Environmental Engineering, The University of Auckland, Auckland, New Zealand

**Keywords:** Hexazinone, UV/H_2_O_2_, Adsorption, Kinetics, Isotherms, Mechanisms

## Abstract

Hexazinone, a globally applied broad-spectrum triazine herbicide, has not been mechanistically investigated previously under advanced oxidation processes (AOPs) and adsorption on activated carbon. In this study, its fate during UV-based oxidation with/without hydrogen peroxide (H_2_O_2_) and adsorption on coconut shell–based granular activated carbon (CSGAC) in water matrices was investigated. A comparison between various irradiation sources (visible, UVA, UVB, and UVC) revealed the highest degradation rate under UVC. More than 98% degradation of hexazinone was observed under 3 J cm^−2^ UVC fluence in the presence of 0.5 mM H_2_O_2_ at pH 7. Moreover, the degradation rate enhanced significantly with an increase in the initial dosage of H_2_O_2_, UV fluence, and contact time in the UV/H_2_O_2_ process. The rate of degradation was lower using secondary effluent than that of Milli-Q water due to the presence of dissolved organics in wastewater. However, the reactions in both matrices obeyed pseudo-first-order kinetics. The effect of different scavengers, including methanol, potassium iodide (KI), and tert-butyl alcohol (TBA), showed that hydroxyl radicals (^•^OH) played a dominant role in hexazinone degradation in the UV/H_2_O_2_ process. Hexazinone was effectively adsorbed by CSGAC through π-π electron donor–acceptor interactions between hexazinone’s triazine ring and CSGAC’s surface functional groups. The isotherm and kinetic studies showed that the adsorption followed the Freundlich model and pseudo-second-order reaction, respectively, suggesting chemisorption. This study provided mechanistic insights on the removal of hexazinone at the tertiary stage of wastewater treatment or the advanced treatment of wastewater reuse.

**Supplementary Information:**

The online version contains supplementary material available at 10.1007/s11356-022-19205-y.

## Introduction

**Fig. 1:**
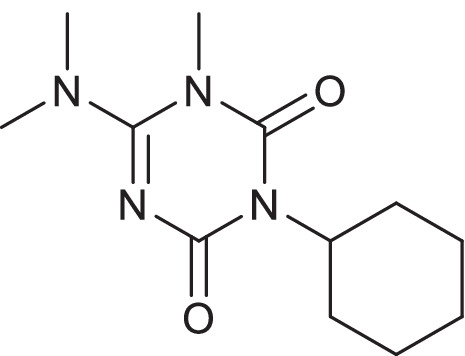
Chemical structure of hexazinone

Over the past few decades, pesticides have been extensively applied in intensive agriculture practices, which has substantially contributed to an increase in the pollution of surface water and groundwater (Adak et al. 2019; Padhye et al. 2013). Hexazinone, a globally used broad-spectrum triazine (Mei et al. 2012; Yao et al. 2019), is considered the most water-soluble triazine herbicide (33 g/L) and is transported to groundwater and surface water near agricultural areas, where it is widely applied (Ganapathy 1996; Martins et al. 2015). It was first registered by the United States Environmental Protection Agency (US EPA) in 1975 for weed control and classified as a group D carcinogen. It can also cause throat, eye, and nose irritation (Martins et al. 2014). It is unsusceptible to photolysis, hydrolysis, and microbial degradation, adding to its persistence in the aquatic environment and resulting in serious environmental concerns (Bouchard et al. 1985; Mei et al. 2012; Yao et al. 2019). Its concentrations in the aquatic environment have been reported to range from ~ 15 ng L^−1^ to 400 µg L^−1^ (Calegari et al. 2018). Figure 1 shows the structure of hexazinone.

Due to the widespread presence of toxic pesticide residues in water resources, more efforts are required to eliminate these contaminants from polluted water (Adak et al. 2019). Advanced oxidation processes (AOPs) and adsorption onto activated carbon (AC) are considered as the most common treatment methods for removal of trace levels of emerging contaminants, including pesticides, from water and wastewater (Kwon et al. 2015; Salman and Hameed 2010). AOPs are considered fast, clean, and effective treatments for the mineralization of contaminants from aqueous solutions. Those rely on the formation of hydroxyl radicals (^•^OH) (Jasemi Zad et al. 2018). However, the complete mineralization of parent compounds may not be possible in AOPs due to the formation of intermediates or degradation products (Agüera and Fernández-Alba 1998). Ultraviolet (UV) irradiation in combination with hydrogen peroxide (H_2_O_2_) is one of the main ^•^OH-based AOPs used for water treatment (Autin et al. 2012; Jazić et al. 2020; Jasemizad et al. 2021). In this process, the organic contaminants can be degraded through direct photolysis, indirect photolysis, and/or oxidation by H_2_O_2_ (Liao et al. 2016; Yao et al. 2013; Jasemizad et al. 2021).

Adsorption onto AC, as the most conventional treatment method, is frequently applied for the removal of pesticides (Adak et al. 2019). Compared to other granular AC, coconut shell–based granular AC (CSGAC) demonstrates a larger adsorption capacity due to its high surface area. This advantage makes it an ideal adsorbent for the adsorption of many organic contaminants from aqueous solutions (Padhye et al 2010).

The photodegradation and photocatalytic degradation of hexazinone have been previously reported in the UV/H_2_O_2_ process under acidic conditions (Martins et al. 2014) and UV/TiO_2_ process (Mei et al. 2012), respectively. However, its fate under AOPs and adsorption on CSGAC has not been mechanistically investigated, especially under environmentally relevant conditions. The aim of this study was, therefore, to investigate the fate of hexazinone during photolysis with UV/H_2_O_2_ and adsorption onto commercial CSGAC. In addition, the effect of different environmental parameters, with a focus on the kinetics and reaction mechanisms, on the removal of hexazinone during advanced oxidation and adsorption was also examined.

## Materials and methods

### Chemicals

Hexazinone was purchased from Sigma-Aldrich, New Zealand. Acetonitrile and methanol (HPLC/MS grade with 99.9% purity), sodium hydroxide (NaOH), H_2_O_2_ (30 wt.% in H_2_O, ACS reagent), ammonium formate (≥ 99% purity, used for the preparation of the aqueous UPLC-MS/MS eluent), tert-butyl alcohol (TBA, 99.5%), and potassium iodide (KI, 99%) were all purchased from Sigma-Aldrich, New Zealand. CSGAC (Acticarb GC1200, 4 × 8 mesh) was from Activated Carbon Technologies, New Zealand. Cellulose acetate syringe filters (0.22 μm) and 5-mL syringes were purchased from Thermo Fisher Scientific, New Zealand. Milli-Q water was obtained from a Millipore Milli-Q® water system (resistivity 18.2 MΩ.cm). All other chemicals used in this study were of analytical grade and commercially available.

### Experimental procedure

A stock solution of hexazinone (1 mM) was prepared in water and stored in the refrigerator (4 °C). The photolysis and adsorption experiments were carried out with 0.5 µM of hexazinone. All batch experiments were conducted at room temperature (25 °C). The secondary wastewater effluent was collected from the Mangere wastewater treatment plant (WWTP), Auckland, New Zealand. The effluent was first filtered through a 0.22 µm membrane and stored at 4 °C prior to use. The effluent samples were spiked with 0.5 µM hexazinone. The pH was adjusted using 0.1 N NaOH and HCl and measured with a pH meter (HACH, HQ40d) calibrated with buffer solutions at pH 4, 7, and 10. Reactions for UV irradiation were carried out from 8 s to 23 min, corresponding to 0.05 to 9 J cm^−2^ UV fluence, respectively. H_2_O_2_ stock with 500 mM initial concentration was daily prepared. Sodium sulfite (100 mM) was added at the end of the reaction to quench the residual H_2_O_2_ (Jasemizad et al. 2021). All experiments were carried out in duplicates. Error bars in all the graphs illustrate the maximum and minimum measurements.

### UV photolysis

All the UV-based experiments were performed in a UV chamber (Opsytec Dr. Grobel, Germany) equipped with eight low-pressure lamps (15 W) (Philips Co., Japan), which emitted light primarily at 254 nm (UVC), 313 nm (UVB), and 352 nm (UVA). Radiometric sensors were used to measure the intensity of each lamp. The UV fluence was calculated using the following equation:

1 $$\mathrm{UV fluence }(\mathrm{mJ }{\mathrm{cm}}^{-2}) =\mathrm{ UV intensity }(\mathrm{mW }{\mathrm{cm}}^{-2}) \times \mathrm{ exposure time }(\mathrm{s})$$

For the UV/H_2_O_2_ process, H_2_O_2_ was added just prior to the irradiation. The sample solutions were continuously mixed with a magnetic stirrer during the experiments.

### Adsorption

In this study, CSGAC was used as an adsorbent to perform the adsorption tests. The characterizations of the adsorbent have been presented in our previous work and are as follows: BET surface area (984 m^2^ g^−1^), micropore volume (0.39 m^3^ g^−1^), mesopore volume (0.029 m^3^ g^−1^), elemental analysis for carbon (85.9%), oxygen (10.7%), hydrogen (0.8%), nitrogen (< 0.3%), sulfur (< 0.3%), and total ash content (2.6%) (Astuti et al. 2021). The adsorbent was first washed with Milli-Q water and then dried at 110 °C in an oven for 24 h (Yu et al. 2016). After being cooled at ambient temperature, the adsorbent was contacted with 100 mL of 0.5 µM hexazinone solutions at pH 7.5. The samples were taken at predetermined intervals of time, filtered, and analyzed for the final concentrations of hexazinone.

The amounts of hexazinone adsorbed (µg g^−1^) at time t (q_t_) and at equilibrium (q_e_) were calculated using the following equations:

2 $${q}_{t}= \frac{\left({C}_{0}-{C}_{t}\right) V}{W}$$

3 $${q}_{e}= \frac{\left({C}_{0}-{C}_{e}\right) V}{W}$$

where *C*_*0*_, *C*_*t*_, and *C*_*e*_ (µg L^−1^) are the concentrations of hexazinone at initial, time *t*, and equilibrium, respectively; *W* (*g*) is the amount of adsorbent used; and *V* (*L*) is the volume of the solution.

The kinetic studies were carried out at different contact times ranging from 2 min to 32 h in the presence of 0.1 g CSGAC. For the isotherm studies, different dosages of CSGAC from 3.12 mg to 0.2 g were used during 24 h contact time.

### Analytical methods and quantification of aqueous hexazinone

The quantification of aqueous hexazinone was performed with liquid chromatography-tandem mass spectrometry (LC–MS/MS) (Shimadzu, Japan). The separation was done on a 2.1 × 100 mm, 3.5 μm, Eclipse Plus C18, Agilent column. The details of LC–MS/MS conditions were described previously (Jasemizad and Padhye 2019).

## Results and discussion

### Photodegradation of hexazinone with and without H_2_O_2_

**Fig. 2:**
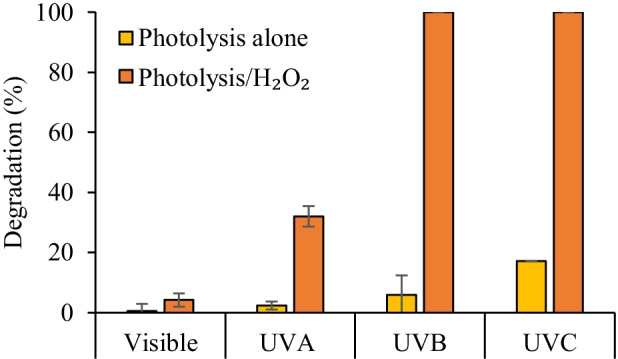
Visible/UV irradiation with and without H_2_O_2_ ([hexazinone]_0_ = 0.5 µM, pH = 7, H_2_O_2_ = 0.5 mM, irradiation fluence for visible light = 14, UVA = 24, UVB = 30, and UVC = 9 J cm^−2^)

The effect of the addition of H_2_O_2_ on the degradation of hexazinone under irradiation by different light sources at neutral pH is shown in Fig. 2. Photolysis alone (no addition of H_2_O_2_) showed a low degradation rate (0.4% to 17%). However, a significant increase in the degradation of hexazinone was observed by the addition of H_2_O_2_, particularly under UVB and UVC (Fig. 2). Visible light did not degrade hexazinone with or without H_2_O_2_. Previously, hexazinone was shown to be resistant to the photolysis alone at pH 2.8 (Martins et al. 2014), but the information under environmentally relevant pH was lacking. The generation of reactive ^•^OH from H_2_O_2_ under irradiation results in the degradation of organic contaminants (Kim et al. 2009; Wang et al. 2017; Jasemizad et al. 2021). Similar results for other pesticides have been reported in the literature. For example, the addition of H_2_O_2_ into the photolysis system resulted in significant degradation of alachlor (Jazić et al. 2020) and 2,4-dichlorophenoxyacetic acid (Adak et al. 2019). Since the highest hexazinone degradation was observed under UVC irradiation, with and without H_2_O_2_, the rest of the experiments were performed under UVC (254 nm).

### The effect of contact time and UV fluence

**Fig. 3:**
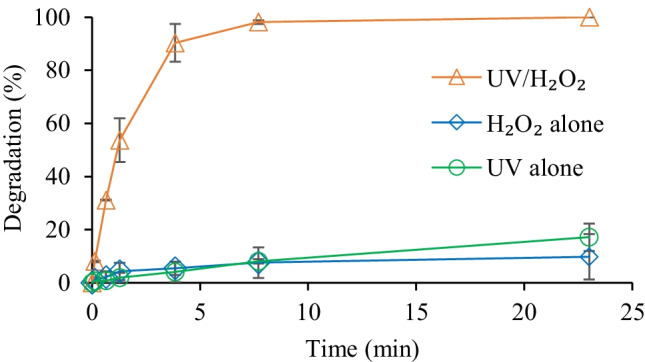
Effect of contact time on degradation of hexazinone ([hexazinone]_0_ = 0.5 µM, pH = 7, H_2_O_2_ dosage = 0.5 mM, UV intensity = 6.5 mJ cm^−2^)

Figure 3 shows the degradation of hexazinone under irradiation and H_2_O_2_ alone and in the UV/H_2_O_2_ process at different contact times from 8 s to 23 min, which corresponded to UV fluence from 0.05 to 9 J cm^−2^. Hexazinone showed low (< 20%) degradation by UV alone and H_2_O_2_ alone. Its degradation increased to 100% by increasing the UV exposure time from 8 s to 7.7 min in the presence of H_2_O_2_ (Fig. 3). In the UV/H_2_O_2_ process, the target compounds are broken down either by irradiation alone (photolysis), by H_2_O_2_, or by ^•^OH (Jasemizad et al. 2021). In the system with H_2_O_2_ or UV irradiation alone, hexazinone degradation was less than 20%, which means the direct photolysis and H_2_O_2_ oxidation alone is impractical for hexazinone degradation (Fig. 3). Hence, the complete degradation of hexazinone in the UV/H_2_O_2_ process is mainly via ^•^OH formation. In this system, two ^•^OH are produced per photon absorbed by H_2_O_2_ at 254 nm (Autin et al. 2012; Martins et al. 2014). These radicals are powerful oxidants, which can rapidly react with many organic contaminants, leading to their effective degradation (Jasemizad et al. 2021).

An increase in the UV intensity in the UV/H_2_O_2_ process results in the greater production of ^•^OH from H_2_O_2_, which are responsible for the mineralization of organic compounds (Jasemizad et al., 2021). Similar results have been observed for other pesticides. An increase in the degradation of alachlor from 50 to about 95% was observed by increasing the UV fluence from 200 to 2000 mJ cm^−2^ in the UV/H_2_O_2_ process at pH 8 in the presence of 0.3 mM H_2_O_2_ (Jazić et al. 2020). In another study conducted by Adak et al. (2019), the degradation of 0.45 mM of 2,4-D increased from 65 to 100% in the same system by increasing the time from 1 to 10 min (UV dose from 100 to 1200 mJ cm^−2^) at pH 4 with 1.125 mM of H_2_O_2_. Almost complete degradation of mecoprop herbicide was observed under 800 mJ cm^−2^ UV fluence and 0.15 mM H_2_O_2_. Metaldehyde pesticide also required 1000 mJ cm^−2^ UV fluence and 0.45 mM H_2_O_2_ for 98% degradation (Semitsoglou-Tsiapou et al. 2016). UV fluence required for > 90% hexazinone removal, observed in this study, is within the range reported in the literature for other pesticides. However, considering the real-world UV fluence is in the range of 40 to 140 mJ cm^−2^ for typical disinfection (Kim et al. 2009) and up to 540 mJ cm^−2^ for control of organic pollutants in wastewater (Kruithof et al. 2007), our results showed that hexazinone would not be removed entirely through commonly practiced AOP parameters at the tertiary stage of wastewater treatment.

### The matrix effect and kinetic studies

**Fig. 4:**
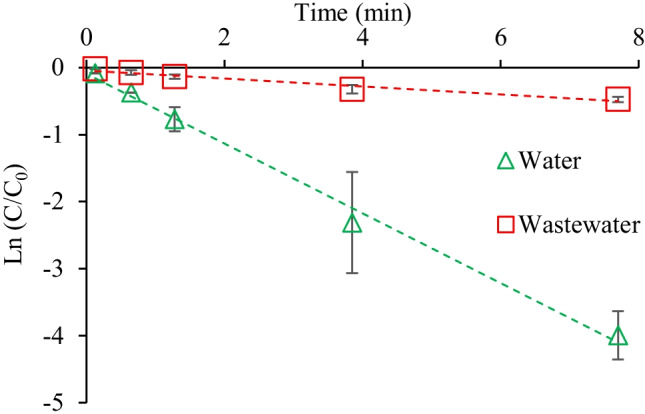
The pseudo-first-order kinetic for hexazinone ([hexazinone]_0_ = 0.5 µM, pH = 7, H_2_O_2_ dosage = 0.5 mM, UV intensity = 6.5 mJ cm^−2^)

Figure 4 shows the degradation rate of hexazinone in the UV/H_2_O_2_ process for Milli-Q water and secondary effluent matrices. The maximum hexazinone removal in the effluent was found to be only 64% at the contact time of 23 min. The decay rate of hexazinone in secondary wastewater effluent (*k* = 0.06 min^−1^) was lower than that in Milli-Q water (*k* = 0.52 min^−1^). This can be explained due to the presence of organic and inorganic compounds in wastewater that can react with ^•^OH and decrease its efficiency for the degradation of individual contaminants (Kumar et al. 2021; Jasemizad et al. 2021).

The k_obs_ (min^−1^) value for photodegradation of hexazinone in both Milli-Q water and wastewater matrices (Eq. 4) was calculated from the plot Ln (C/C_0_) vs time (Fig. 4), where *C*_*0*_ and *C* are the initial concentration of hexazinone and its concentration after degradation at time t (min), respectively.

4 $$\mathrm{Ln}\left(\frac{C}{{C}_{0}}\right)=-{\mathrm{k}}_{\mathrm{obs}}.\mathrm{t}$$

H_2_O_2_:hexazinone molar ratio in this study’s UV/H_2_O_2_ experiments was 1000:1; hence, the K_obs_ can be assumed to be independent of H_2_O_2_ and ^•^OH concentrations. The straight lines obtained for hexazinone degradation in Fig. 4, with regression coefficient (*R*^2^) > 0.96, confirmed that the degradation of hexazinone in the UV/H_2_O_2_ process followed the pseudo-first-order reaction kinetics. In the literature, some other pesticides were also shown to follow the pseudo-first-order degradation kinetics for the UV/H_2_O_2_ treatment (Oancea and Meltzer 2014; Ulu 2019; Castro-Narváez et al. 2020; Li et al. 2011; Jazić et al. 2020; Adak et al. 2019). The pseudo-first-order kinetic was also suitable for modelling hexazinone’s degradation in the UV/TiO_2_ process (Mei et al. 2012).

### The effect of varying H_2_O_2_ dosages

**Fig. 5:**
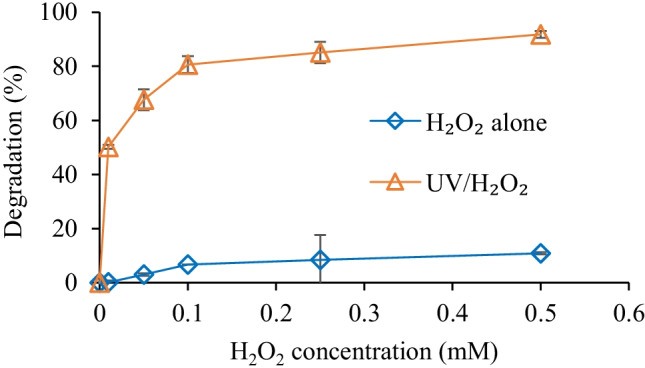
Effect of the H_2_O_2_ dosage on degradation of hexazinone ([hexazinone]_0_ = 0.5 µM, pH = 7, UV fluence = 1.5 J cm^−2^, H_2_O_2_ alone contact time = 30 min, UV/H_2_O_2_ process contact time = 3.8 min)

Figure 5 shows the effect of different concentrations of H_2_O_2_ with/without UV irradiation. As evident from Fig. 5, the degradation of hexazinone increased from 50 to 92% (k_H2O2_ = 3.29 mM^−1^) by increasing the dosage of H_2_O_2_ from 0.01 to 0.5 mM under 1,500 mJ cm^−2^ UV fluence. This increase in the degradation of hexazinone in the presence of a higher concentration of H_2_O_2_ under UV irradiation can be explained by the absorption of more UV photons by a higher amount of H_2_O_2_, which results in the generation of more radicals. Similar results have been reported in the literature. For example, an increase in the degradation rate of tartrazine, with 10.35 µM initial concentration, was observed by increasing the concentrations of H_2_O_2_ from about 0.1 to 0.4 mM. In that study, researchers found that by increasing the concentration of H_2_O_2_ to 0.67 mM, the degradation of tartrazine decreased and was lower than 0.4 mM H_2_O_2_ (Oancea and Meltzer 2014). High concentrations of H_2_O_2_ have been shown to cause a negative effect on the degradation rate of target compounds, which could be owing to the scavenging impact of H_2_O_2_ on ^•^OH (Li et al. 2011; Wang et al. 2017). However, since the concentrations of H_2_O_2_ used in the present study were below the scavenging points reported in the literature, such a decline in degradation was not observed in this study. Moreover, our results confirmed that H_2_O_2_ alone is not suitable for the degradation of hexazinone, with the highest observed degradation of only 11%.

### The effect of pH

**Fig. 6:**
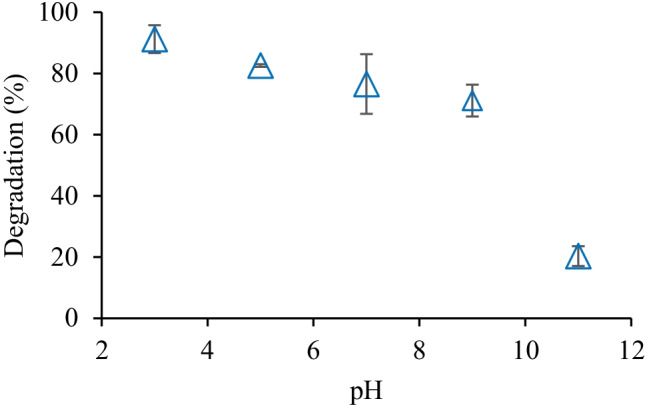
Effect of pH ([hexazinone]_0_ = 0.5 µM, H_2_O_2_ dosage = 0.5 mM, UV fluence = 1 J cm^−2^)

The initial pH of the aqueous solution exhibits different effects on the reactivity of contaminants and, hence, the overall efficiency of the AOPs. In this study, the effect of various pHs (3–11) on hexazinone degradation was assessed at 0.5 mM of H_2_O_2_ and 1 J cm^−2^ of UV fluence. As shown in Fig. 6, hexazinone degradation was highest (91%) at acidic pH and lowest (20%) at basic pH. Hexazinone has a pKa of 2.2 and was neutral for the tested pH range. It is reported that in the neutral form, molecules have strong light absorption and high photochemical reactivity, which results in high removal efficiency (Borowska et al. 2015). The decrease in the degradation of hexazinone under alkaline conditions can be explained by two competing processes: the generation of ^•^OH and the scavenging of ^•^OH (Sharma et al. 2015). The formation of hydroperoxide anion (HO_2_^−^) (Eq. 5), which is favorable at higher pH values, can enhance the generation of ^•^OH in the UV/H_2_O_2_ process (Eq. 6). On the other hand, HO_2_^−^ can also act as a scavenger of ^•^OH (Eq. 7) with a faster reaction rate than H_2_O_2_ (Eq. 8). Furthermore, H_2_O_2_ loses its characteristics as an oxidant due to its reaction with HO_2_^−^ (Eq. 9) (Narayanasamy and Murugesan 2014; Xiao et al. 2016).

5 $${\mathrm{H}}_{2}{\mathrm{O}}_{2}\leftrightarrow {\mathrm{HO}}^{-}_{{2}}+{\mathrm{H}}^{+}(\mathrm{pKa}=11.6)$$

6 $${\mathrm{HO}}^{-}_{{2}}+{\mathrm{H}}^{+}\to 2{}^{\bullet }\mathrm{OH}$$

7 $${\mathrm{HO}}^{-}_{{2}}+{}^{\bullet }\mathrm{OH}\to {\mathrm{HO}}_{2}^{\bullet }+\mathrm{OH}^{-}(k=7.5\times {10}^{9}{\mathrm{M}}^{-1}{\mathrm{S}}^{-1})$$

8 $${\mathrm{H}}_{2}{\mathrm{O}}_{2}+{}^{\bullet }\mathrm{OH}\to {\mathrm{HO}}_{2}^{\bullet }+{\mathrm{H}}_{2}\mathrm{O}(k=2.7\times {10}^{7}{\mathrm{M}}^{-1}{\mathrm{S}}^{-1})$$

9 $${\mathrm{HO}}^{-}_{{2}}+{\mathrm H}_2{\mathrm O}_2\rightarrow{\mathrm H}_2\mathrm O+{\mathrm O}_2+\mathrm{HO}^{{}^-}$$

### The effect of scavengers

**Fig. 7:**
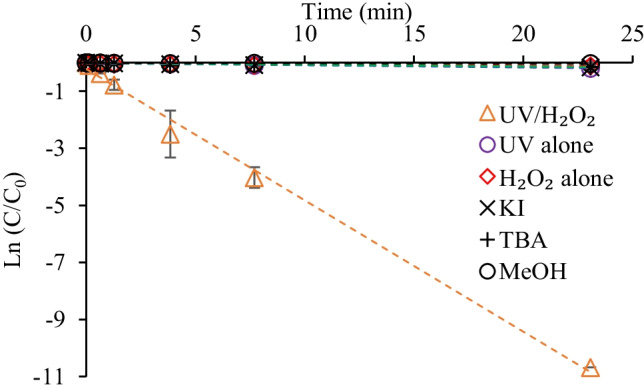
Hexazinone degradation kinetics in UV/H_2_O_2_ process ([hexazinone]_0_ = 0.5 µM, pH = 7, H_2_O_2_ dosage = 0.5 mM, scavengers Conc. = 0.1 M, UV intensity = 6.5 mW cm^−2^)

The effect of 0.1 M of methanol, TBA, and KI, known ^•^OH scavengers (Kumar et al. 2021; Jasemizad et al. 2021), in the UV/H_2_O_2_ experiments was evaluated in this study to assess the contribution of ^•^OH to hexazinone degradation. As can be seen from Fig. 7, the photodegradation of hexazinone in the UV/H_2_O_2_ process was significantly hindered by the addition of radical scavengers. The degradation rate of hexazinone in the presence of KI (*k* = 0.007 min^−1^) and TBA (*k* = 0.006 min^−1^) was almost similar as H_2_O_2_ alone (*k* = 0.004 min^−1^). In addition, methanol (*k* = 0.001 min^−1^) showed the highest inhibitory effect on hexazinone degradation in the UV/H_2_O_2_ process (Fig. 7 and Fig. S2). Results demonstrated the important role of ^•^OH in hexazinone degradation during AOPs. Ran et al. (2014) had previously demonstrated that TBA could effectively scavenge the ^•^OH in the ozone/H_2_O_2_ process for hexazinone degradation.

### Proposed photodegradation pathways in the presence of hydrogen peroxide

The identification of the intermediate by-products of hexazinone photodegradation during the UV/H_2_O_2_ process was carried out by LC–MS/MS using the MS full scan and spectra (Fig. S3). The oxidation of 10 µM hexazinone was conducted at various irradiation times under 6.5 mW cm^−2^ UVC irradiation in the presence of 0.1 mM H_2_O_2_ at neutral pH. The identified photodegradation by-products from hexazinone were *m/z* 267 and 239, which were previously identified for photocatalytic degradation of hexazinone in the UV/TiO_2_ process (Mei et al. 2012). Although smaller intermediates (*m/z* 241, 223, 147, 102) were not detected in the present study, the possible photodegradation of hexazinone is expected to be similar to the pathway presented by Mei et al. (2012), due to the dominant ^•^OH degradation pathway even for the current study. In Mei et al. study, about 20 µM hexazinone was completely degraded within 40 min of exposure to UV irradiation in the presence of TiO_2_ under neutral pH. Yao et al. (2019) identified a few more intermediates from 70 min electrochemical degradation of hexazinone, with ^•^OH as a primary oxidant. Those included *m/z* 305, 301, 267, 261, 255, 253, 241, 239, 237, 225, 223, 217, 91, 74, and 60.

### Adsorption of hexazinone on CSGAC

**Fig. 8:**
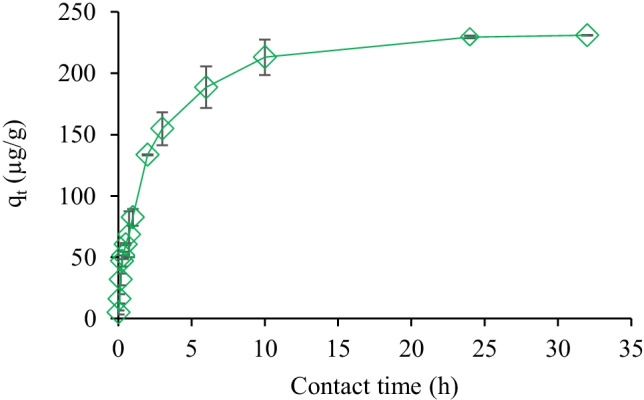
Effect of contact time on the adsorption rate of hexazinone (0.5 µM) onto 0.1 g CSGAC at pH 7.5

The adsorption of hexazinone on CSGAC was performed at different contact times, demonstrating an improvement in the removal of hexazinone with time. The adsorption enhanced from 2 to 64% by increasing the time from 2 min to 3 h, and then gradually reached equilibrium at 24 h (Fig. 8) with ~ 95% adsorption. The higher adsorption at the initial stages of the process can be due to the larger surface area available on CSGAC. At the later stages of the adsorption, the pores of the adsorbent get saturated with hexazinone molecules. Furthermore, there are repulsive forces between the molecules in the solution and the molecules on CSGAC at the later stages (Njoku et al. 2014; Salman et al. 2011), which lowers the adsorption rate.

### Adsorption kinetics

Adsorption kinetics are strongly related to the physical and/or chemical properties of the adsorbent (Tan et al. 2015). To understand the adsorption mechanism of hexazinone on CSGAC, the pseudo-first-order (Eq. 10) and pseudo-second-order (Eq. 11) kinetic models (Jasemizad et al. 2021) were studied (Fig. S4).

10 $$\mathrm{Ln }\left({\mathrm{q}}_{\mathrm{e}}-{\mathrm{q}}_{\mathrm{t}}\right)=\mathrm{Ln }{\mathrm{q}}_{\mathrm{e}}- {\mathrm{k}}_{1}\mathrm{t}$$

11 $$\frac{\mathrm{t}}{{\mathrm{q}}_{\mathrm{t}}}= \frac{1}{{\mathrm{k}}_{2}{\mathrm{q}}_{\mathrm{e}}^{2}}+\frac{1}{{\mathrm{q}}_{\mathrm{e}}}\mathrm{ t}$$

where *k*_1_ (min^−1^) and *k*_2_ (g mg^−1^ min^−1^) are the rate constants of pseudo-first-order and the pseudo-second-order reactions, respectively, and *t* (min) is the contact time. The kinetic model parameters are listed in Table 1. The pseudo-second-order model with *R*^2^ > 0.99 best describes the adsorption kinetics of hexazinone onto CSGAC. Moreover, the agreement between the experimental q_e_ value and the calculated q_e_ value in pseudo-second-order kinetic confirmed the suitability of this kinetic model. It suggested that the adsorption rate depended more on the availability of the adsorption sites on the adsorbent rather than hexazinone concentration in the solution (Njoku et al. 2014; Salman et al. 2011). A similar observation has been reported for the adsorption of some pesticides onto GAC (Salman and Hameed 2010), coconut fronds AC (Njoku et al. 2014), and banana stalk AC (Salman et al. 2011). Furthermore, the pseudo-second-order kinetic suggested that the rate-limiting step is controlled by chemisorption, resulting in the chemical bonding between hexazinone molecules and the functional groups on the surface of CSGAC (Tan et al. 2015).

**Table 1:** Comparison of the pseudo-first-order and pseudo-second-order adsorption rate constants

C_0_ (µg L^**−**1^)	^*^q_e exp_ (µg g^**−**1^)	Pseudo-first order			Pseudo-second order		
		^*^q_e cal_ (µg g¯^1^)	*k*_1_ (min^−1^)	*R*^2^	q_e cal_ (µg g﻿¯^1^)	*K*_2_ (g mg^−1^ min^−1^)	*R*^2^
240.16	229	187.2	0.005	0.982	243	0.046	0.998

*q_e__exp_ and q_e__cal_ (μg g−1) are experimental and model-predicted adsorption capacity at time *t*

### Adsorption isotherms

To better understand the adsorption behavior of hexazinone onto CSGAC, two typical isotherm models, Freundlich (Eq. 12) and Langmuir (Eq. 13), were investigated (Table 2 and Fig. S5) (Foo and Hameed 2010).

**Table 2:** Sorption isotherm parameters of hexazinone onto CSGAC

Freundlich			Langmuir			
*K*_*f*_ (µg g^−1^) (L µg^−1^)^1/n^	*n*	*R*^2^	*K*_*L*_ (L µg^−1^)	*R*_*L*_	*q*_*m*_ (µg g^−1^)	*R*^2^
62.52	2.03	0.987	0.041	0.165	714.3	0.95

12 $${q}_{e}= {K}_{f}{C}_{e}^{{~}^{1}\!\left/ \!\!{~}_{n}\right.}$$

13 $${q}_{e}= \frac{{q}_{m}{K}_{L}{C}_{e}}{1+ {k}_{L}{C}_{e}}$$

where *q*_e_ is the amount of hexazinone adsorbed per g of CSGAC (μg g^−1^) and *C*_e_ is the equilibrium concentration (μg L^−1^). K_f_ and K_L_ are the Freundlich constant (μg g^−1^) (L μg^−1^)^1/n^, and the Langmuir constant (L μg^−1^), respectively. The parameter q_m_ is the maximum adsorption capacity (μg g^−1^), and *n* is a measure of adsorption linearity.

The adsorption of hexazinone onto CSGAC was best described by the Freundlich model with the highest *R*^2^, suggesting that the adsorption of hexazinone onto CSGAC involves multi-layer adsorption with interactions of the adsorbates’ molecules with the heterogeneous surface of adsorbent (Foo and Hameed 2010; Salman et al. 2011). Moreover, this can be confirmed by the *n* values (*n* > 1) in the Freundlich model, representing higher intensity and favorability for chemisorption (Foo and Hameed 2010; Sumaraj et al. 2020).

### Proposed adsorption mechanism

The mechanism of hexazinone adsorption could be attributed to the interactions between CSGAC surface functional groups, confirmed through FTIR analysis, and hexazinone’s triazine ring through π-π electron donor–acceptor interactions. The adsorption of several pesticides onto activated carbon cloth had been attributed to the dispersion forces between the π electrons in pesticide structure and π electrons in the adsorbent. Moreover, the aromatic ring in the structure of the pesticides was assumed to enhance the probability of such interactions owing to the delocalization of π electrons over the ring (Ayranci and Hoda 2005).

## Conclusions

In this study, the performance of the UV irradiation with/without H_2_O_2_ and adsorption on CSGAC for the removal of hexazinone in aqueous solutions was examined. The results indicated that the photodegradation rate of hexazinone could be enhanced by the addition of H_2_O_2_ in the presence of UV, particularly under UVC irradiation, due to the formation of more ^•^OH from H_2_O_2_. However, UV and H_2_O_2_ individually were not effective for hexazinone degradation. Moreover, an increase in the irradiation time, UV fluence, and H_2_O_2_ concentration could significantly improve the degradation rate. The photodegradation of hexazinone in the UV/H_2_O_2_ process followed the pseudo-first-order kinetic reactions. The effect of ^•^OH scavengers, including methanol, KI, and TBA, confirmed the significant role of ^•^OH in the degradation of hexazinone during AOPs. Our results show that hexazinone removal in real-world AOP conditions could be problematic, considering the low levels of oxidants used as well as the presence of other organics in secondary effluents which scavenge ^•^OH. However, adsorption could be an effective treatment for hexazinone removal. Almost complete adsorption of hexazinone was achieved in the presence of 0.1 g of CSGAC within 24 h. The adsorption kinetic and isotherm models of hexazinone fitted the pseudo-second-order and Freundlich curves, respectively. The main adsorption mechanism of hexazinone on CSGAC was found to be chemisorption. The findings of the study provide helpful information to researchers and practitioners regarding hexazinone removal at advanced stages of wastewater treatment and/or reuse.

## Supplementary Information

Below is the link to the electronic supplementary material.

Supplementary file1 (DOCX 135 KB)

## Data Availability

Not applicable.
