# Supplementary material for: Photodegradation and adsorption of hexazinone in aqueous solutions: removal efficiencies, kinetics, and mechanisms
Source: Environ Sci Pollut Res Int. 2022 Feb 21;29(32):48330–9. doi: 10.1007/s11356-022-19205-y (PMC9252970; doi:10.1007/s11356-022-19205-y)
Supplement: Supplementary file 1 — Supplementary file1 (DOCX 135 KB) [file 11356_2022_19205_MOESM1_ESM.docx]

**Supporting Information**

**Journal of Environmental Science and Pollution Research**

**Photodegradation and Adsorption of Hexazinone in Aqueous Solutions: Removal Efficiencies, Kinetics, and Mechanisms**

**Tahereh Jasemizad^1^ and Lokesh P. Padhye^1,^***

^1^Department of Civil and Environmental Engineering, The University of Auckland, Auckland, New Zealand

*Corresponding Author:

Phone: +64 9 923 2410; E-mail: [l.padhye@auckland.ac.nz](mailto:l.padhye@auckland.ac.nz)

Number of pages: 5

Number of Figures: 5

**Fig S1.** Effect of deionized water and secondary effluent matrices on degradation of hexazinone ([hexazinone]_0_= 0.5 µM, pH=7, H_2_O_2_ dosage= 0.5 mM, UV intensity= 6.5 mJ cm^-2^)

**Fig S2.** Effect of scavengers in UV/H_2_O_2_ process on degradation of hexazinone ([hexazinone]_0_= 0.5 µM, pH=7, H_2_O_2_ dosage= 0.5 mM, scavengers Conc.= 0.1 M, UV intensity= 6.5 mJ cm^-2^).

**Fig S3.** LC-MS/MS spectra of oxidation of hexazinone in UV/H_2_O_2_ process

**Fig S4.** The pseudo-first order (a) and pseudo-second order (b) kinetics for adsorption of hexazinone

**Fig S5.** The a) Freundlich, and b) Langmuir isotherms for adsorption of hexazinone onto CSGAC
